# Incidence of Cancer in Patients with Irritable Bowl Syndrome

**DOI:** 10.3390/jcm10245911

**Published:** 2021-12-16

**Authors:** Sven H. Loosen, Markus S. Jördens, Mark Luedde, Dominik P. Modest, Simon Labuhn, Tom Luedde, Karel Kostev, Christoph Roderburg

**Affiliations:** 1 Clinic for Gastroenterology, Hepatology and Infectious Diseases, University Hospital Düsseldorf, Medical Faculty of Heinrich Heine, University Düsseldorf, 40225 Düsseldorf, Germany; markus.joerdens@med.uni-duesseldorf.de (M.S.J.); simon.labuhn@med.uni-duesseldorf.de (S.L.); Christoph.Roderburg@med.uni-duesseldorf.de (C.R.); 2 KGP Bremerhaven, 27574 Bremerhaven, Germany; mark.luedde@web.de; 3 Department of Hematology, Oncology and Tumorimmunology, Charité-Universitätsmedizin Berlin, Freie Universität Berlin, Humboldt-Universität zu Berlin, and Berlin Institute of Health, 10117 Berlin, Germany; dominik.modest@charite.de; 4 German Cancer Consortium (DKTK), German Cancer Research Centre (DKFZ), 69120 Heidelberg, Germany; 5 Epidemiology, IQVIA, 60549 Frankfurt, Germany; Karel.Kostev@iqvia.com

**Keywords:** IBS, cancer, tumor, functional disorder, gastrointestinal

## Abstract

(1) Background: Irritable bowel syndrome (IBS) represents one of the most common disorders of gut–brain interaction (DGBI). As recent data has suggested an increased cancer incidence for IBS patients, there is an ongoing debate whether IBS might be associated with a risk of cancer development. In the present study, we evaluated and compared incidence rates of different malignancies including gastrointestinal cancer in a large cohort of outpatients, with or without IBS, treated in general practices in Germany. (2) Methods: We matched a cohort of 21,731 IBS patients from the IQVIA Disease Analyzer database documented between 2000 and 2019 in 1284 general practices to a cohort of equal size without IBS. Incidence of cancer diagnoses were evaluated using Cox regression models during a 10-year follow-up period. (3) Results: In 11.9% of patients with IBS compared to 8.0% without IBS, cancer of any type was diagnosed within 10 years following the index date (*p* < 0.001). In a regression analysis, this association was confirmed in female (HR: 1.68, *p* < 0.001) and male (HR = 1.57, *p* < 0.001) patients as well as in patients of all age groups. In terms of cancer entity, 1.9% of patients with and 1.3% of patients without IBS were newly diagnosed with cancer of digestive organs (*p* < 0.001). Among non-digestive cancer entities, the strongest association was observed for skin cancer (HR = 1.87, *p* < 0.001), followed by prostate cancer in men (HR = 1.81, *p* < 0.001) and breast cancer in female patients (HR = 1.80, *p* < 0.001). (4) Conclusion: Our data suggest that IBS might be associated with cancer of the digestive organs as well as with non-digestive cancer entities. However, our findings do not prove causality and further research is warranted as the association could be attributed to life style factors that were not documented in the database.

## 1. Introduction

Irritable bowel syndrome (IBS) is one of the most common gastrointestinal disorders, representing a major health and socioeconomic burden worldwide [1]. The clinical presentation of IBS is complex and varies widely between individuals [1]. Accordingly, the definition of IBS has evolved over time and different diagnostic systems and criteria have been proposed [1]. According to the current S3 guideline [2], irritable bowel syndrome (IBS) disease is present when all of the following three items are met: (1) there are chronic symptoms, i.e., lasting longer than three months (e.g., abdominal pain, flatulence), which are referred to the intestine by the patient and the physician and are usually accompanied by changes in bowel movements; (2) the complaints should justify the patient seeking help and/or worrying about them and be so severe that quality of life is relevantly impaired as a result; (3) there are no characteristic alterations for other clinical pictures which are probably responsible for these symptoms. In addition, the international expert working group just recently updated the diagnostic criteria for IBS (Rome IV criteria) [3,4].

The pathophysiology of IBS is multifactorial and not fully elucidated yet. Different studies suggested that—at least in subgroups of patients—IBS is associated with activation of the immune system [5,6]. This immune activation may maintain a low-grade inflammation, which results in an continuously increased release of proinflammatory cytokines [7]. Of note, elevated serum levels of CCL28 were observed in patients with irritable bowel syndrome [8]. This cytokine has recently been associated with inflammatory diseases and cancer in humans [9,10]. Based on these data, as well as on observational data suggesting increased cancer incidences in IBS patients [11], there is an ongoing debate on whether IBS is associated with risk of cancer development [11,12].

In this study we therefore compared the incidence of different cancer entities in patients with IBS to a matched cohort of patients without IBS.

## 2. Materials and Methods

### 2.1. Database

This study was based on data from the Disease Analyzer database (IQVIA), which contains drug prescriptions, diagnoses, and basic medical and demographic data obtained directly and in anonymous format from computer systems used in the practices of general practitioners and specialists [13]. The database covers approximately 3% of all outpatient practices in Germany. Diagnoses (according to International Classification of Diseases, 10th revision (ICD-10)), prescriptions (according to Anatomical Therapeutic Chemical (ATC) Classification system), and the quality of reported data are being monitored by IQVIA. In Germany, the sampling methods used to select physicians’ practices are appropriate for obtaining a representative database of general and specialized practices. It has previously been shown that the panel of practices included in the Disease Analyzer database is representative of general and specialized practices in Germany [13]. This database has already been used in previous studies focusing on digestive system diseases [14], as well as cancer [15,16].

### 2.2. Study Population

**Figure 1:**
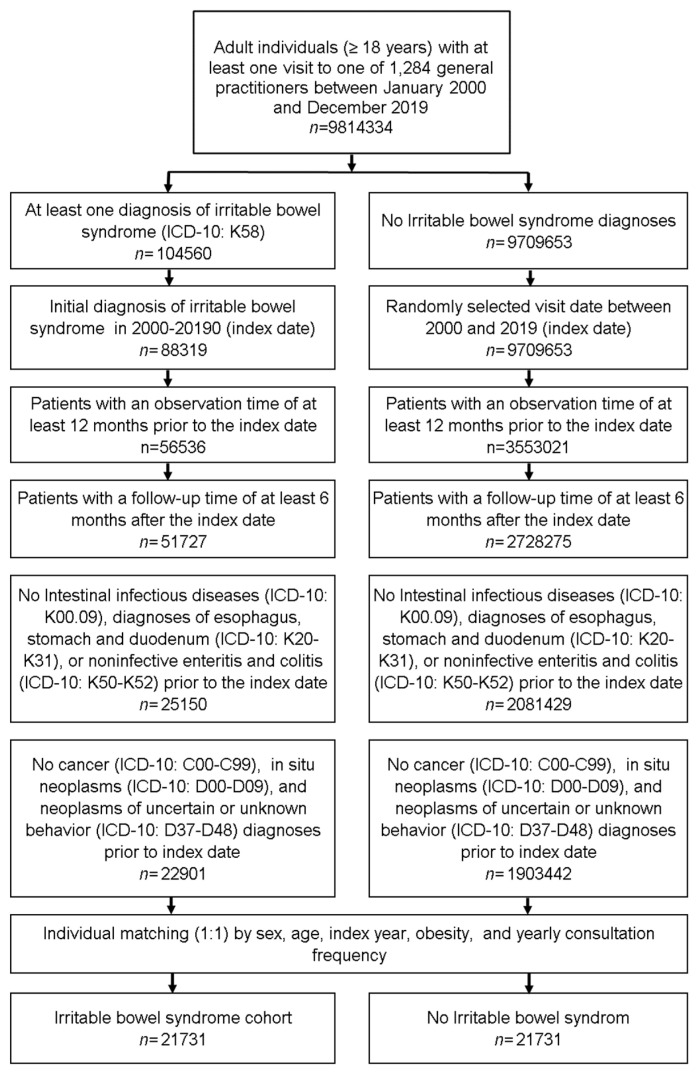
Selection of study patients.

This retrospective cohort study included adult patients (≥18 years) with an initial diagnosis of irritable bowel syndrome (ICD-10: K58) in 1284 general practices in Germany between January 2000 and December 2019 (index date; Figure 1). An inclusion criterium was an observation time of at least 12 months prior to the index date and a follow-up time of at least six months after the index date. To identify the correct IBS diagnosis, patients with intestinal infectious diseases (ICD-10: K00.09), diagnoses of esophagus, stomach and duodenum (ICD-10: K20-K31), or noninfective enteritis and colitis (ICD-10: K50-K52) diagnoses within 12 months prior to the index date were excluded. Patients with cancer diagnoses (ICD-10: C00-C99), in situ neoplasms (ICD-10: D00-D09), and neoplasms of uncertain or unknown behavior (ICD-10: D37-D48) prior to index date were excluded to enable the investigation of the cancer incidence after the diagnosis of IBS.

IBS patients were matched to non-IBS patients by sex, age, index year, obesity and yearly consultation frequency. As IBS patients have much higher consultation frequency by GPs due to IBS investigations and treatment, and higher consultation frequency can increase the probability of other diagnoses documentation, we included consultation frequency per year in the matching. For the non-IBS patients, the index date was that of a randomly selected visit between January 2000 and December 2019 (Figure 1).

### 2.3. Study Outcomes and Covariates

The main outcome of the study was the incidence of cancer (ICD 10: C00-C99) in total, and cancer of different organs, including digestive organs (ICD 10: C15-C26), respiratory organs (ICD 10: C30-C39), skin (ICD 10: C43, C44), breast (ICD 10: C50), male genital organs (ICD 10: C60-C63), urinary tract (ICD 10: C64-C68), and lymphoid and hematopoietic tissue (ICD 10: C81-C96) as a function of IBS.

### 2.4. Statistical Analyses

Differences in the sample characteristics between patients with and without irritable bowel syndrome were tested using McNemar tests for categorical variables and paired Wilcoxon tests for continuous variables. Cox regression models were conducted to study the association between the IBS and cancer incidence. These models were performed separately for different cancer entities. To counteract the problem of multiple comparisons, *p*-values < 0.01 were considered statistically significant. Analyses were carried out using SAS version 9.4 (SAS institute, Cary, NC, USA).

## 3. Results

### 3.1. Basic Characteristics of the Study Sample

The present study included 21,731 patients diagnosed with IBS and 21,731 patients without IBS. The basic characteristics of study patients are summarized in Table 1. Mean age (SD) was 48.4 (17.3) years; 67.2% of patients were female, 6.7% of patients were obese. The average yearly GP consultation frequency was 3.7 (SD: 4.1) times during the follow-up time.

### 3.2. IBS Is Associated with an Increased Incidence of Cancer

**Figure 2:**
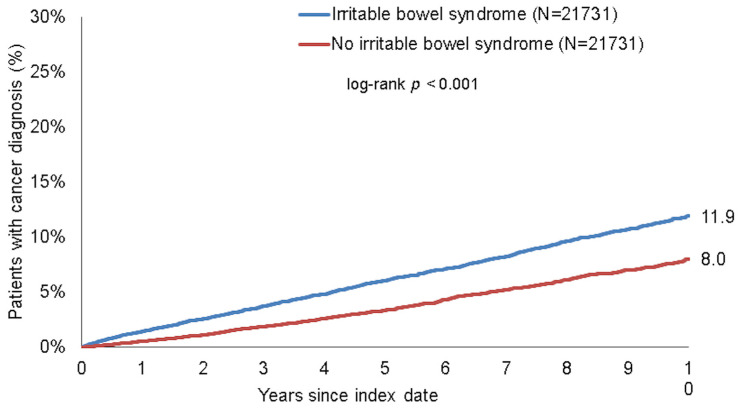
Kaplan–Meier curves for time to cancer diagnosis in patients with and without IBS.

Within 10 years of the index date, 11.9% of patients with IBS and 8.0% of those without IBS were diagnosed with cancer (log-rank *p* < 0.001, Figure 2). In Cox regression analyses, IBS was significantly associated with the incidence of cancer (HR: 1.64, 95%CI: 1.51–1.78, *p* < 0.001 in total, HR: 1.68, 95%CI: 1.52–1.87, *p* < 0.001 in women, HR = 1.57, 95%CI: 1.37–1.80, *p* < 0.001 in men). Moreover, a significant association was found for all age groups (Table 2). 

### 3.3. Association between IBS and Cancer of Digestive Organs

**Figure 3:**
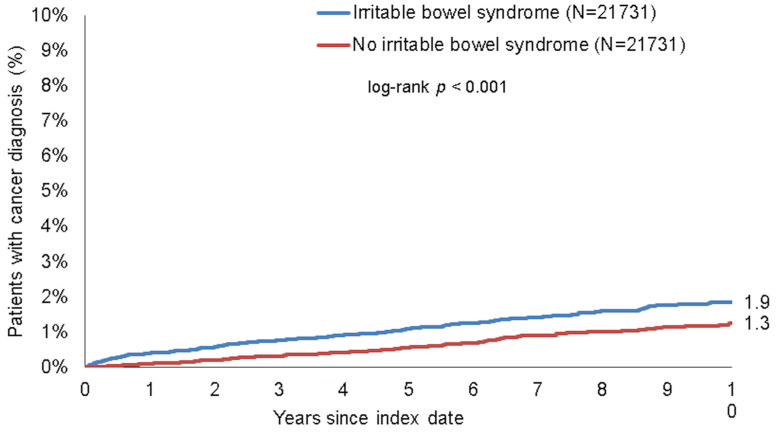
Kaplan–Meier curves for time to digestive organ cancer diagnosis in patients with and without IBS.

**Figure 4:**
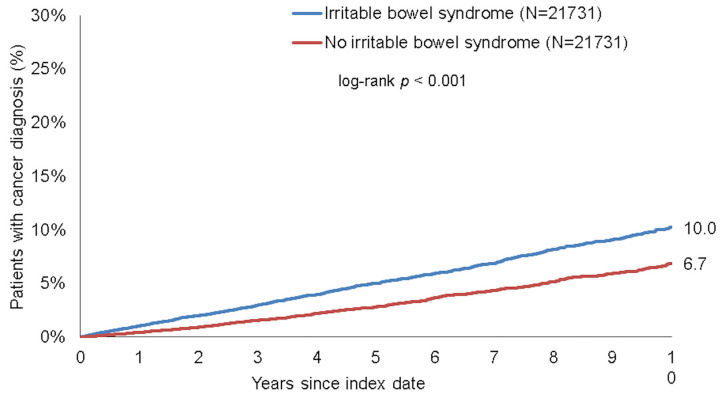
Kaplan–Meier curves for time to non-digestive organ cancer diagnosis in patients with and without IBS.

To further dissect the association between IBS and new cancer diagnoses, we subsequently compared incidence rates of different specific cancer sites between patients with or without IBS. Within 10 years of the index date, 1.9% of patients with IBS and 1.3% of those without IBS were newly diagnosed with cancer of digestive organs (log-rank *p* < 0.001, Figure 3). These proportions were 10.0% vs. 6.7% for non-digestive organ cancers (log-rank *p* < 0.001, Figure 4). Results were confirmed in regression analysis, revealing a significant association between IBS and cancer of digestive organs (HR: 1.75, 95%CI: 1.42–2.14, *p* < 0.001), as well as non-digestive organ cancer (HR: 1.62, 95%CI: 1.48–1.78, *p* < 0.001, Table 3). Interestingly, among the non-digestive cancer entities, the strongest association was observed for skin cancer (HR = 1.87, *p* < 0.001), followed by prostate cancer in men (HR = 1.81, *p* < 0.001) and breast cancer in female patients (HR = 1.80, *p* < 0.001, Table 3). There were no significant associations between IBS and respiratory organ cancers nor with urinary tract cancer (Table 3).

## 4. Discussion

By analyzing a large cohort of 21,731 IBS patients from the IQVIA disease analyzer database who were followed in generalized practices in Germany, we demonstrate that the incidence of cancer is higher in patients with IBS as compared to a matched cohort. Notably, this effect was independent of patient gender or age.

IBS is a common disease with variable manifestations, affecting up to 10% of the population and often occurs in episodes [17]. Gastrointestinal infections might be a trigger of symptom onset, but the exact mechanisms of disease development/activation are not fully understood [18]. Recently an overlap of IBS and other gastrointestinal diseases including cancer has been suggested [11]. However, data on this association are scarce, highlighting the need for further studies. In addition to gastrointestinal cancers, we demonstrate a significant increase in various cancer entities including skin cancer, prostate cancer and breast cancer. We therefore hypothesized that the mechanism potentially leading to cancer in IBS patients is independent of pure local inflammation of the intestinal mucosa, although immune reaction still seems to mostly occur in the intestine [19]. A general systemic inflammatory response, as shown for inflammatory bowel disease, might be an explanation for the generally increased tumor incidence [7]. One exploratory reason for this observation could be an alteration of the gut microbiome, as recently suggested in IBS patients [20]. Notably, in many patients with IBS an increase in *Streptococcus spp*. or *Clostridium perfringens* and a decrease in *Bacteroides* was detected [21]. Thus, there is an overall increase in pathogenic species and displacement of beneficial species, but the findings are not consistent with other studies [22,23]. In this context, several studies linked alteration of the gut microbiome to low-grade inflammation of the bowel mucosa, challenging the traditional view of IBS as a purely functional disorder [24].

In tumorigenesis of colorectal carcinoma, alterations of the microbiome have been described [25]. Several studies suggested that specific alterations in the microbiome, as seen in patients with IBD, may be a risk factor for the development of colorectal neoplasia. For example, *Streptococcus bovis* or *gallolyticus* in stool of patients are clearly associated with colorectal tumorigenesis [26,27,28,29]. For stool-*Streptococcus bovis* it is even suggested to favor development of extraintestinal cancer [30]. Similar observations have been made for esophageal carcinoma, with emphasis on the inflammation of the mucosa by specific bacterial species [31]. In line with these findings, we found an association of IBS with extraintestinal tumor diagnoses. Changes in the microbiome associated to cancerogenesis were also observed for prostate carcinoma and breast cancer [32,33] and could explain our findings as increased incidence of prostate cancer or breast cancer appears to be associated with IBS. Furthermore, chronic inflammation and activation of the immune system may promote extraintestinal tumorigenesis, e.g., gynecological tumors like endometrial, cervical or ovarian cancer [34]. At least in a subgroup of IBS patients, an increased cytokine release was found to be connected to a low-grade inflammatory reaction [7]. In particular, increased serum levels of CCL28 seem to be of relevance here, as there is an association with tumor diseases in humans [8,9]. Therefore, the combination of altered microbiome and chronic inflammatory processes in IBS patients might be a driver of tumorigenesis not just in digestive organs, but outside of the gastrointestinal tract as well.

In our population, the incidence of GI cancers, unlike non-GI cancers, increased in the first months after IBS diagnosis. A likely explanation might be that in some cases GI cancer symptoms may have been falsely interpreted as IBS in the initial diagnostic algorithm and turned out to be cancer after a short follow-up interval. These data highlight that if “red flags” (e.g., anemia, positive fecal occult blood test (FOBT) are present, or fecal calprotectin), gastrointestinal symptoms require thorough clarification by endoscopy to avoid misinterpretation of complaints as functional disorders. 

Nevertheless, our study has important limitations due to its design, which are unavoidable due to the nature of retrospective database analysis. First, misclassifications of diagnoses within the ICD-10 coding system may have occurred and cannot be corrected since no monitoring or source data verification were implemented. This point is particularly relevant, as the diagnostic criteria of IBS have changed during the study period (Rome III vs. IV). Second, the database does not include information on potential confounders such as patients’ lifestyle including alcohol consumption and smoking behavior, socioeconomic status, some important laboratory values, TNM status, hospital data, and mortality information. Therefore, patient matching for these potential confounders was not possible and any conclusion that IBS itself is causally associated with cancer development must be drawn carefully. In addition, as this database only covers approximately 3% of outpatient practices, findings might not represent the total population. However, GP consultations in Germany were shown to be representative within the database. Nevertheless, our data and data from other groups support future controlled observational studies to better quantify the risk of tumor disease in IBS patients and to confidently rule out any bias in the observed association. In this context, the need for systematic cancer screening in IBS patients should be reevaluated based on these future trials and respective screening recommendations could be made.

## 5. Conclusions

Our analyses suggest an increased risk of intestinal but also extraintestinal tumors in patients with IBS.

## Figures and Tables

**Table 1:** Basic characteristics of the study sample (after 1:1 matching by sex, age, index year, obesity, and yearly consultation frequency).

Variable	Proportion Affected among Patients with IBS (%) N = 21,731	Proportion affected among Patients without IBS (%)N = 21,731	*p*-Value
Age (Mean, SD)	48.4 (17.3)	48.4 (17.3)	1.000
Age 18–40	34.8	34.8	1.000
Age 41–50	20.1	20.1	
Age 51–65	26.4	26.4	
Age > 65	18.7	18.7	
Women	67.2	67.2	1.000
Men	32.8	32.8	
Obesity	6.7	6.7	1.000
Yearly consultation frequency	3.7 (4.1)	3.7 (4.1)	1.000

Proportions of patients in % given, unless otherwise indicated. SD: standard deviation.

**Table 2:** Association between irritable bowel syndrome and the incidence of cancer diagnoses in patients followed in general practices in Germany (Cox regression models).

Cohort	Proportion Affected among Patients with IBS (%)	Proportion Affected among Patients without IBS (%)	HR (95% CI)	*p* Value
Total	11.9	8.0	1.64 (1.51–1.78)	<0.001
Women	11.4	7.4	1.68 (1.52–1.87)	<0.001
Men	13.0	9.3	1.57 (1.37–1.80	<0.001
Age 18–40	4.0	2.5	1.77 (1.36–2.29)	<0.001
Age 41–50	8.9	6.3	1.48 (1.21–1.81)	<0.001
Age 51–65	14.7	10.6	1.63 (1.42–1.86)	<0.001
Age > 65	24.4	17.0	1.69 (1.41–1.84)	<0.001

**Table 3:** Association between irritable bowel syndrome and the incidence of different cancer diagnoses in patients followed in general practices in Germany (Cox regression models).

Cancer Site	Proportion Affected among Patients with IBS (%)	Proportion Affected among Patients without IBS (%)	HR (95% CI)	*p* Value
Digestive organs	1.9	1.3	1.75 (1.42–2.14)	<0.001
No digestive organs	10.0	6.7	1.62 (1.48–1.78)	<0.001
Respiratory organs	0.7	0.6	1.17 (0.85–1.61)	0.327
Skin	3.0	1.9	1.87 (1.55–2.24)	<0.001
Female breast (women)	2.9	1.7	1.80 (1.45–2.24)	<0.001
Prostate (men)	3.2	1.9	1.81 (1.33–2.45)	<0.001
Urinary tract	0.6	0.5	1.25 (0.87–1.78)	0.225
Lymphoid and hematopoietic tissue	1.6	1.0	1.40 (1.12–1.75)	0.003

## Data Availability

Data are available upon request from the Department of Gastroenterology, Hepatology and Infectious Diseases of the University Hospital Düsseldorf for researchers who meet the criteria for access to confidential data: Wissenschaft.Gastro@med.uni-duesseldorf.de.
